# Analysis of Clinicopathological Characteristics and Prognosis of Carcinosarcoma of the Breast

**DOI:** 10.1155/2022/3614979

**Published:** 2022-07-01

**Authors:** Yao Tian, Xiaofeng Liu, Yingxi Li, Qingxiang Meng, Yumian Jia, Baichuan Wang, Xianghui He

**Affiliations:** ^1^Department of General Surgery, Tianjin Medical University General Hospital, Tianjin 300052, China; ^2^The First Department of Breast Cancer, Tianjin Medical University Cancer Institute and Hospital, National Clinical Research Center for Cancer, Tianjin 300060, China; ^3^Key Laboratory of Breast Cancer Prevention and Therapy, Ministry of Education, Tianjin Medical University Cancer Institute and Hospital, National Clinical Research Center for Cancer, Tianjin 300060, China; ^4^Key Laboratory of Cancer Prevention and Therapy, Tianjin Medical University Cancer Institute and Hospital, Tianjin's Clinical Research Center for Cancer, Tianjin 300060, China; ^5^Department of Pathogen Biology, School of Basic Medical Sciences, Tianjin Medical University, Tianjin 300070, China; ^6^Department of Radiotherapy, Tianjin Medical University Cancer Institute and Hospital, National Clinical Research Center for Cancer, Tianjin 300060, China; ^7^Department of Breast Cancer Pathology and Research Laboratory, Tianjin Medical University Cancer Institute and Hospital, National Clinical Research Center for Cancer, Tianjin 300060, China; ^8^Anhui Medical University Clinical College of Chest, Hefei 230022, Anhui Province, China; ^9^Anhui Chest Hospital, Hefei 230022, Anhui Province, China

## Abstract

**Background:**

Few cases of carcinosarcoma of the breast have been reported because of its low incidence rate and rapid progression. Seeking effective therapeutic methods becomes urgent in clinical practice. This study was aimed to investigate the clinical characteristics of carcinosarcoma of the breast and to explore proper therapeutic methods for patients with this rare tumor.

**Methods:**

We conducted a retrospective analysis on 47 patients with carcinosarcoma of the breast receiving treatment in our hospital from 2003 to 2020. Most of these patients received primary surgery followed by adjuvant chemotherapy, while four patients had lumpectomy only. Statistics showed no preference in age and menopausal status of patients.

**Results:**

The overall survival rate and progression-free survival rate of all patients at a median follow-up time of 33 months were 63.8% and 57.4%, respectively. Tumor size at diagnosis and chemotherapy strategies were both significant prognostic factors in reference to disease-free survival (DFS) and overall survival (OS) of the patients (tumor size: *p*=0.023 for DFS and *p*=0.021 for OS; therapeutic method: *p*=0.041 for DFS and *p*=0.024 for OS). N stage at diagnosis was significant only with reference to overall survival of the patients (*p*=0.009). EGFR expression was positive in some patients.

**Conclusions:**

Our results elucidated that the patients received comprehensive therapy, especially adjuvant chemotherapy was indispensable for better outcomes. Early detection and treatment were necessary for a higher survival rate when the tumor size was less than 5 cm without lymph node metastasis. Prospective outcomes with novel strategies targeting EGFR need to be further investigated.

## 1. Background

Carcinosarcoma of the breast (CSB) is an extraordinarily rare disease, accounting for 0.08–0.2% of all breast malignancies. It was firstly reported by Virchow in 1864 [1, 2]. CSB was referred to as a type of metaplastic carcinoma by the World Health Organization in 2003 [3]. As a highly heterogenous disease, CSB is usually observed with two completely different cell lines containing breast ductal carcinoma component and sarcoma-like component, which is often defined as a tumor with mixed histological characteristics containing both epithelial and mesenchymal tissues without a transitional zone [2, 4–6]. Loss of intercellular adhesion is a key characteristic of CSB, exhibiting downregulation of epithelial markers and upregulation of mesenchymal-related proteins. Meanwhile, the immunoreactivity of vimentin, actin, and S-100 was also confirmed in previous investigations [5, 7, 8].

However, the histogenesis of CSB is still controversial. The spindle cells, cystosarcoma phyllodes, preexisting fibroadenoma, and cystic microenvironment are thought to contribute to the origin of carcinosarcoma in previous reports [9–12]. Due to the remarkably low incidence of CSB, neither basic research nor clinical studies on therapeutic outcomes and prognosis have been defined. Until now, the prognosis and optimal therapy of CSB is not clear enough to generate standard guidelines. In comparison with patients with classical types of breast cancer, patients with CSB are believed to achieve a worse prognosis because of its lower differentiation degree in tumor cells, higher histological grade, and aggressiveness [2, 13–15]. There are still some other reports suggesting no difference in the survival rate of CSB and that of other “typical” invasive breast tumors [16].

To present a more precise and comprehensive summary of the characteristics of CSB, we retrospectively studied 47 patients with CSB in our hospital and further correlated demographic features and clinical information with therapeutic outcomes and prognosis of the patients. We hope our investigations can provide significant evidences for the standard treatment towards carcinosarcoma of the breast in the future.

## 2. Methods

### 2.1. Patients

47 patients diagnosed with CSB from 2003 to 2020 in our hospital were retrospectively analyzed. Clinical information was collected from their medical records, including demographic characteristics, surgery methods, pathological and immunohistochemical results, TNM stages, subsequent treatment strategies, follow-up data, and prognosis. The study was approved by the IRB of hospital. Relevant investigations were conducted according to the principles stated in the Declaration of Helsinki.

### 2.2. Diagnosis

All reviewed patients were diagnosed to have CSB by two or more senior pathologists in our hospital. The most important characteristic of CSB was defined as a tumor histologically containing both epithelial and mesenchymal elements without a transitional zone between them [2, 4–6]. Positivity of an estrogen receptor (ER) or progesterone receptor (PR) was defined as more than 1% of tumor cells with positive nuclear staining by immunohistochemistry. Human epidermal growth factor receptor 2 (HER2/neu) was analyzed according to the criteria of the American Society of Clinical Oncology (ASCO)/the College of American Pathologists (CAP). Intensity patterns with scores 0 to 1+ were defined as negative and 3+ as positive; while those scored as 2+ were suggested to be further evaluated with fluorescent *in situ* hybridization (FISH).

### 2.3. Treatment Strategies

According to the standard treatment of breast cancer formulated in our hospital, patients underwent surgery first and were then diagnosed. Of the total forty-seven patients, the majority (44/47, 93.6%) of patients received mastectomy as their primary therapeutic method; while only three patients received neoadjuvant chemotherapy prior to operation. 6 patients received radical mastectomy and 37 patients received modified radical mastectomy. The remaining 4 patients had lumpectomy and refused further treatment.

The chemotherapy regimens mainly consisted of administration of cyclophosphamide, docetaxel, and other antineoplastic agents. Two patients received cyclophosphamide, methotrexate, and 5-fluorouracil, comprising the CMF regimen. 6 patients adopted epirubicin and docetaxel, comprising the TE regimen. Docetaxel and cisplatin (regimen TP) were recommended to 3 patients. 10 patients turned to an induction backbone of docetaxel, epirubicin, and cyclophosphamide, comprising the TEC regimen. One patient received cyclophosphamide, epirubicin, and cisplatin (regimen CEP). Courses were recommended to be repeated every 3 weeks.

### 2.4. Follow-Up

Patients were followed up every 3 months after the completion of treatment for the first two years. B-scan ultrasonography, mammography, and blood examination were performed to evaluate the efficacy of the treatment. Follow-up visit was conducted every 6–12 months after treatment from the third year. By October 1^st^, 2020, all 47 patients were followed up. Disease-free survival (DFS) was calculated from the time of diagnosis until disease progression. Overall survival (OS) was calculated from the time of diagnosis and death for any reason or the last contact with the patient if no event occurred.

### 2.5. Immunohistochemistry

Immunohistochemistry of tissues from patients with CSB was conducted according to the standard protocol of the Department of Breast Cancer Pathology and Research Laboratory in our hospital. In brief, formalin-fixed and paraffin-embedded (FFPE) sections of CSB tissues were subjected to immunostaining with primary antibodies of 34*β*E12, CK8/18, estrogen receptor, progesterone receptor, HER2, vimentin, and EGFR. 2-*μ*m thick tissue sections were deparaffinized, rehydrated and subjected to antigen retrieval by boiling in sodium citrate buffer (10 mM, pH 6.0). The sections were incubated at 4°C overnight with primary antibody at 1 : 100 dilution and then stained with 3,3′-diaminobenzidine. After visualization of immunoreactivity, the sections were counterstained with hematoxylin and mounted. Adjacent noncancerous tissues were used as internal controls. The staining results were determined as follows: positive when immunoreactivity was observed and negative when immunoreactivity was absent.

### 2.6. Statistical Analysis

The statistical software SPSS 22.0 was used to analyze the collected data. The *χ*^*2*^ test was used to assess categorical variables and the Student's independent *t*-test to compare continuous variables. Cumulative survival analysis was performed by the Kaplan–Meier method, and the log-rank test was used for single-factor analysis. All *p* values were two-tailed, and *p* < 0.05 was considered statistically significant.

## 3. Results

### 3.1. Patient Characteristics

Demographic characteristics of the patients are recorded in Table 1. All 47 patients included in this study, ranging from 22 to 83 years old, were female, and the median age was 53 years old. A palpable lump without pain was the most common symptom. Tumor size was determined by a postoperative pathological inspection or maximum diameter of computerized tomography (CT) if neoadjuvant chemotherapy was performed. The maximum size at the time of diagnosis ranged from 1 to 10 cm (mean 3.7 cm and median 3.5 cm). 35 patients (35/47, 74.5%) had a tumor smaller than 5 cm, while 12 patients (12/47, 25.5%) had larger than 5 cm. Lymph node metastasis occurred in 8 patients (8/47, 17.0%). According to the staging system of the American Joint Committee on Cancer, more than 70% of (T1: 9/47, 19.1%; T2: 26/47, 55.3%) patients were in the early stage of breast malignancy. 9 patients (9/47, 19.1%) were diagnosed at stage I, 33 patients (33/47, 70.2%) at stage II, and 5 patients (5/47, 10.6%) at stage III. Of the patients with an obtainable hormone receptor state, there were no patients exhibiting ER positive, 3 patients (3/47, 6.4%) with PR positive, and only one patient (1/47, 2.1%) with HER2/neu positive. Epidermal growth factor receptor (EGFR) was available in only 20 patients for lack of accurate data, and 16 patients (16/20, 34%) were positive.

### 3.2. Pathological Features

**Figure 1:**
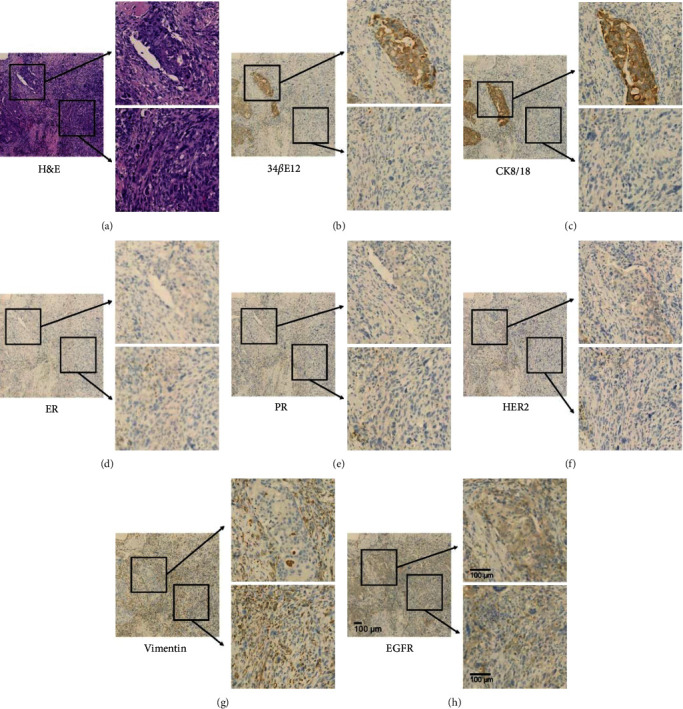
H&E staining (a) and immunohistochemical images (indicating 34*β*E12, CK8/18, estrogen receptor, progesterone receptor, HER2, vimentin, and EGFR from (b) to (h), respectively) of a representative patient with CSB. Main images were captured at 100× magnification, with two regional magnifications indicating the carcinoma area at the upper right and sarcoma area at lower right.

H&E images and immunohistochemistry results of a representative patient with CSB are shown in Figure 1. Considering the particularity of CSB tissue components, we selected the visual field which included both carcinoma and sarcoma tissues to comprehensively determine the overall expression of each protein, as well as to identify the difference between the carcinoma tissue and sarcoma tissue.

According to the results, we can conclude that the main immunohistochemical characteristics of CSB were positive in CK and 34*β*E12, while negative in ER, PR, and HER2. The expression of vimentin was positive in sarcoma components, while negative in other adjacent tissue components, indicating a heterogenous status of CSB. In addition, EGFR was positively expressed in both cancerous and sarcoma components, indicating that targeting EGFR may be effective in inhibiting the proliferation of both carcinoma and sarcoma components.

### 3.3. Prognosis of the Patients

**Figure 2:**
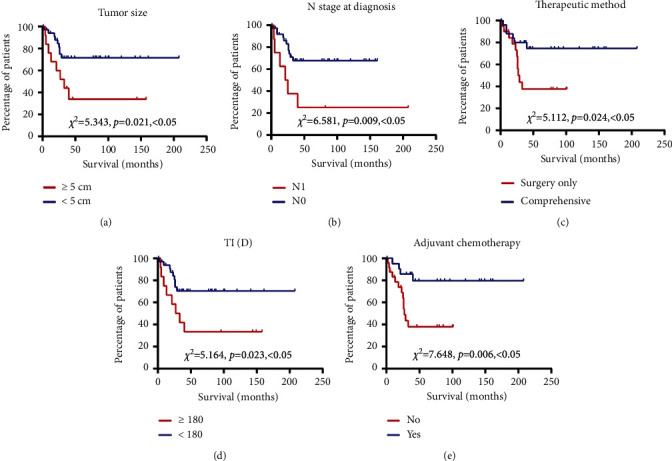
Kaplan–Meier survival curves showing overall survival of patients with CSB according to (a) tumor size, (b) N stage at diagnosis, (c) therapeutic method, (d) TI and (e) adjuvant chemotherapy.

**Figure 3:**
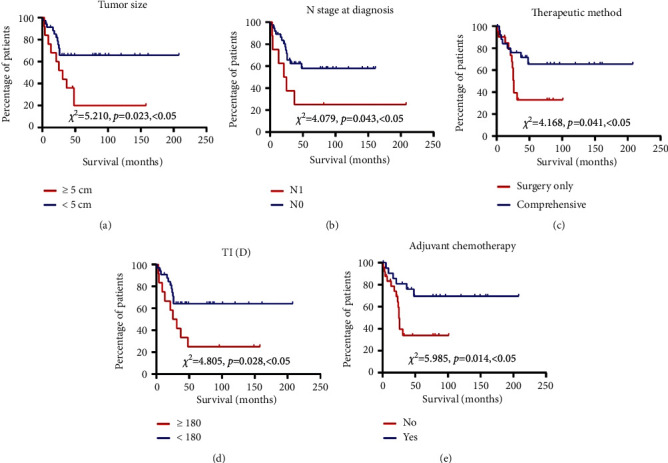
Kaplan–Meier survival curves showing disease-free survival of patients with CSB according to (a) tumor size, (b) N stage at diagnosis, (c) therapeutic method, (d) TI, and (e) adjuvant chemotherapy.

Up to the terminal date of this study, 30 patients were alive while three relapsed but lived with breast cancer. 17 patients had died of palindromia. With a median follow-up time of 33 months (range 1–208 months) for patients, the OS rate and PFS rate were 63.8% and 57.4%, respectively (Figures 2 and 3).

The information of components in the tumor was available in 8 patients. Of these, the epithelial components composed of infiltrative ductal carcinoma occurred in 7 patients and adenocarcinoma in 1 patient. Mesenchymal component in 2 patients turned to be chondrosarcoma, 3 fibroblastic, 2 osteoblastic, and 1 fatty sarcoma.

According to the univariate analysis shown in Tables 2 and 3, DFS of patients was significantly affected by the tumor size (*p*=0.023), therapeutic method (*p*=0.041), TI (time interval between first discomfort and first consultation) (*p*=0.028), and adjuvant chemotherapy (*p*=0.014). The OS rate of patients was significantly affected by the tumor size (*p*=0.021), N stage at diagnosis (*p*=0.009), therapeutic method (*p*=0.024), TI (*p*=0.023), and adjuvant chemotherapy (*p*=0.006). According to the above data, patients whose tumor size was larger than 5 cm with lymph node metastasis had a poor prognosis. Patients who received comprehensive therapy, especially adjuvant chemotherapy, had better outcomes in both DFS and OS, indicating that adjuvant chemotherapy was a significant predictor in the prognosis of patients. However, univariate analysis showed that the age, menopausal status, and *T* stage at diagnosis did not have an impact on both OS and DFS of patients.

Based on the Cox proportional hazards regression model, we found that TI and adjuvant chemotherapy are independent prognostic factors of DFS in patients with carcinosarcoma. However, in terms of OS of patients with CSB, independent prognostic factors are TI, N stage at diagnosis, and adjuvant chemotherapy.

## 4. Discussion

The morbidity of carcinosarcoma of the breast is rare. Most investigations about the clinical features and therapeutic methods of CSB are sporadic case reports at present. Characterized by the inexistent region of the transitional edge between the epithelial and mesenchymal elements, this aggressive tumor often presents as high-grade, poorly differentiated large lumps with negative lymph node and hormone receptors [14,17]. Owing to a larger cohort of patients, our study provides some details on its characteristics and therapeutic methods.

Given the exiguity of CSB, its origin is still in dispute. Myoepithelial cells, myofibroblastic metaplasia, preexisting fibroadenomas, and phyllodes tumors were considered contributing to the tumorigenesis in previous reports [5, 12, 18–20]. Myoepithelial source theory prevailed because of its biopotential differentiation capacity [20]. Pathologically, previous studies have demonstrated that the epithelial components in CSB may range from infiltrative ductal carcinoma to adenocarcinoma, squamous carcinoma, alveolar cell carcinoma, or *in situ* carcinoma. In terms of morphology, the mesenchymal components may manifest as undifferentiated sarcoma, malignant fibrous histiocytoma, chondrosarcoma, or osteosarcoma [21–23]. Consistent with previous reports, we found that the most significant composition of the tumor obtained was infiltrative ductal carcinoma and chondrosarcoma.

Compared with other classic types of breast cancer, the median tumor size of CSB is usually larger, which varies between 3.5 and 5.3 cm [24–26] at the first visit. While larger median tumor diameter was reported in some individual studies [14]. In parallel with the previous studies, the median diameter of tumors enrolled in our study was 3.50 cm. According to univariate analysis in our patients, tumor size is a vital predictor of OS (*p*=0.021, tumor size <5 cm *vs*. ≥5 cm) and DFS (*p*=0.023, tumor size <5 cm *vs.* ≥5 cm). This result is similar to that in the earlier study by Wargotz ES et al. which clarified the relationship between tumor size and death or progression [5]. When classified by *T* stage (T1 vs. T2, T3, and T4), it indicated that *T* stage at diagnosis had no impact on OS (*p*=0.217) and DFS (*p*=0.097) in our study. However, in a report from MD Anderson, *T* stage and survival rate were strongly correlated [17]. This difference may correlate with the race of the enrolled patients, as the main statistics of our study were Chinese patients, while the investigation conducted by MD Anderson enrolled Caucasian patients.

Because of the rarity of CSB and its lack of effective systemic management, there exists a disagreement about the 5-year survival rate in the published literature, which varies from 49–68% [25]. A detailed study revealed that OS was stage-related and the 5-year OS rate at stage I, II, III, and IV was 0.73, 0.59, 0.44, and 0, respectively. Esses KM [16] reported that the survival rate was not statistically different between CSB and invasive breast cancer, but the prognosis was reported to be poorer in most studies due to its aggressiveness and recurrence [1,13,14]. The survival rates in our patients were better than those in the preceding reports, where the 5-year OS rate and DFS rate were 72.2% and 56.3%, respectively. Probably early diagnosis and the average small tumor size contributed to this result.

The optimal method for CSB therapy is unclear. Currently, the remedy of CSB is to a great extent similar to that of classic breast carcinoma. Due to the invasiveness and sarcomatous path, mastectomy is commonly performed [27]. Modified radical mastectomy (MRM) was recommended in the majority of previous reports [12–14], especially for patients with T2 or higher stage disease [17]. In our study, only 4 patients received lumpectomy and half of them relapsed. At the last follow-up visit, 15 patients receiving MRM had recurrences. Whether MRM is safe and effective for CSB is inconclusive. Consistent with other reports, CMF chemotherapy and anthracycline/taxol-based therapy occupied the major choice in adjuvant chemotherapy of CSB and the latter was reported to be more valid [6,12]. However, because of the rare cases, we could not prove which one was more effective.

Nowadays, most of the studies have addressed the role of the inhibitors of epidermal growth factor receptor (EGFR) for the potential treatment of CSB [28, 29]. In a previous study, amplification and overexpression of EGFR gene occurred in 14 out of 20 patients suffering from metaplastic carcinomas (MCS) [28], but whether patients can benefit from targeted drugs such as gefitinib or cetuximab should be further identified. In our study, the same small series, 10 out of 17 patients with available EGFR expression results were positive, but none of them received gefitinib or cetuximab. Despite the lack of data, we hypothesized that targeted drugs are safe and effective towards CSB. Accordingly, we would recommend novel therapeutic methods combining EGFR-targeting drugs in the future.

## 5. Conclusion

Our study elucidated that carcinosarcoma of the breast is clinically aggressive with poor prognosis mainly because of the high probability of recurrence. Patients who received comprehensive treatment, especially adjuvant chemotherapy, achieved better outcomes. Early detection and treatment were indispensable for a higher survival rate when the tumor size was less than 5 cm and without lymph node metastasis. As EGFR was positively expressed in several patients with CSB, novel therapeutic strategies targeting EGFR will be a promising method for the treatment of CSB, which need to be further investigated.

## Figures and Tables

**Table 1:** Clinicopathological features and treatment modalities in patients with carcinosarcoma of the breast.

Characteristics	Number of patients	%
Age		
≤50	21	44.7
>50	26	55.3
Menopausal status		
Premenopausal	22	46.8
Postmenopausal	25	53.2
TI (D)		
<180	35	74.5
≥180	12	25.5
Tumor size		
<5 cm	35	74.5
≥5 cm	12	25.5
*T* stage at diagnosis		
T1	9	19.1
T2	26	55.3
T3	10	21.3
T4	2	4.3
N stage at diagnosis		
N0	39	83.0
N1	8	17.0
Estrogen-receptor status		
Positive	0	0.0
Negative	46	97.9
Unknown	1	2.1
Progesterone-receptor status		
Positive	3	6.4
Negative	43	91.5
Unknown	1	2.1
HER2/neu (IHC and/or FISH) status		
Positive	1	2.1
Negative	45	95.7
Unknown	1	2.1
EGFR status		
Positive	16	34.0
Negative	4	8.5
Unknown	27	57.5
Therapeutic method		
Surgery only	21	44.7
Comprehensive therapy	26	55.3
Breast surgery		
Radical mastectomy	6	12.8
Modified radical mastectomy	37	78.7
Lumpectomy	4	8.5
Neoadjuvant chemotherapy		
Yes	3	6.4
No	44	93.6
Adjuvant chemotherapy		
Yes	22	46.8
No	25	53.2
Adjuvant radiation therapy		
Yes	4	8.5
No	43	91.5
Adjuvant hormonal therapy		
Yes	0	0.0
No	47	100.0

TI: time interval between first discomfort and first consultation; HER2: epidermal growth factor receptor 2; IHC: immunohistochemistry; FISH: fluorescent *in situ* hybridization technique; EGFR: epidermal growth factor receptor.

**Table 2:** Univariate and multivariate analysis of patients' disease-free survival.

Factors	DFS			
	Univariate		Multivariate	
	*p* value	HR (95% CI)	*p* value	HR (95% CI)
Age	0.120	2.015 (0.832–4.878)		
Menopausal status	0.151	1.914 (0.790–4.638)		
Tumor size	0.023	3.362 (1.187–9.524)		
T stage at diagnosis	0.097	0.363 (0.110–1.200)		
N stage at diagnosis	0.043	3.663 (1.039–12.91)		
Therapeutic method	0.041	0.385 (0.154–0.963)		
TI (D)	0.028	3.164 (1.130–8.862)	0.005	3.752 (1.499–9.392)
Adjuvant chemotherapy	0.014	0.324 (0.132–0.800)	0.004	4.396 (1.587–12.173)

HR: hazard ratio; CI: confidence interval; TI (D): time interval between first discomfort and first consultation (days).

**Table 3:** Univariate and multivariate analysis of patients' overall survival.

Factors	OS			
	Univariate		Multivariate	
	*p* value	HR (95% CI)	*p* value	HR (95% CI)
Age	0.416	1.490 (0.571–3.891)		
Menopausal status	0.552	1.337 (0.514–3.479)		
Tumor size	0.021	3.780 (1.224–11.67)		
T stage at diagnosis	0.217	0.457 (0.132–1.585)		
N stage at diagnosis	0.009	6.427 (1.596–25.89)	0.008	4.844 (1.511–15.526)
Therapeutic method	0.024	0.320 (0.119–0.859)		
TI (D)	0.023	3.674 (1.196–11.29)	0.023	3.296 (1.177–9.227)
Adjuvant chemotherapy	0.006	0.254 (0.096–0.671)	0.001	0.123 (0.035–0.425)

HR: hazard ratio; CI: confidence interval; TI (D): time interval between first discomfort and first consultation (days).

## Data Availability

The datasets used and/or analyzed during the current study are available from the corresponding authors on reasonable request.

## Abbreviations

CSB: Carcinosarcoma of the breast
DFS: Disease-free survival
EGFR: Epidermal growth factor receptor
ER: Estrogen receptor
FFPE: Formalin-fixed paraffin-embedded
HER2: Human epidermal growth factor receptor 2
MCS: Metaplastic carcinomas
MRM: Modified radical mastectomy
OS: Overall survival
PR: Progesterone receptor.
